# Color adaptation induced from linguistic description of color

**DOI:** 10.1371/journal.pone.0173755

**Published:** 2017-03-30

**Authors:** Liling Zheng, Ping Huang, Xiao Zhong, Tianfeng Li, Lei Mo

**Affiliations:** School of Psychology, Center for Studies of Psychological Application and Key Laboratory of Mental Health and Cognitive Science of Guangdong Province, South China Normal University, Guangzhou, P.R. China; University of Akron, UNITED STATES

## Abstract

Recent theories propose that language comprehension can influence perception at the low level of perceptual system. Here, we used an adaptation paradigm to test whether processing language caused color adaptation in the visual system. After prolonged exposure to a color linguistic context, which depicted red, green, or non-specific color scenes, participants immediately performed a color detection task, indicating whether they saw a green color square in the middle of a white screen or not. We found that participants were more likely to perceive the green color square after listening to discourses denoting red compared to discourses denoting green or conveying non-specific color information, revealing that language comprehension caused an adaptation aftereffect at the perceptual level. Therefore, semantic representation of color may have a common neural substrate with color perception. These results are in line with the simulation view of embodied language comprehension theory, which predicts that processing language reactivates the sensorimotor systems that are engaged during real experience.

## Introduction

Two families of theories have proposed contrasting ways of how language is processed in the brain in relation to the sensorimotor systems. In the traditional symbolic view, language processing is based on abstract, amodal symbols [1,2,3], and people gain the meaning of language through the relations among symbols. From this point of view, the mind is an abstract information processor and sensorimotor systems are not relevant to language processing. In contrast, theories of embodied language comprehension propose a direct link between language and sensorimotor systems. They propose that language is understood by constructing mental simulations of the objects and events being described, and these simulations involve the re-activation of sensory, motor, and affective systems that are engaged during real experience [4,5,6,7]. In this account, language processing recruits, to some extent, sensorimotor systems. Thus, embodied semantics predict that comprehension should interact with perception processing, as they recruit the same sensorimotor system.

A large number of empirical studies have been claimed to support the embodied semantics. In neuroscience, researchers have found that the same sensorimotor regions of the brain are activated when individuals process words and their referents [8,9,10]. In cognitive psychology, many studies [11,12,13,14] have provided evidence for an interaction between comprehension and visual processing, which could be separated into two categories: the priming of perception by language [14] and the priming of language by perception [15]. In this line of research, priming paradigm was most commonly used. For example, participants were presented with a picture after reading a sentence, the content of which could match or mismatch with the picture [16]. The task was to determine whether the picture item had been mentioned in the previous sentence. The results indicated that participants were faster to respond in the match than in the mismatch condition. The match advantage can be viewed as providing support for the embodied view because it suggests that sensory representations are routinely activated during verbal comprehension [17].

However, some researchers have argued that the interaction between language and perception could also be explained by symbolic semantics. For example, Pavan and Baggio [18] proposed that in the match-mismatch paradigm, linguistic stimuli largely activated the semantic network, as required by symbolic semantics, and that the pictures were rapidly analyzed also at the semantic level, where a comparison or integration with the verbal material was carried out. Another criticism came from Mahon and Caramazza [19], who thought that although the previous studies did demonstrate that the motor and sensory systems were activated, they did not demonstrate that the activation of motor or sensory information constituted the semantic analysis of the sentence. From their point of view, the interference could be occurring at a decision-making stage after critical semantic analysis.

The controversy between the embodied view and the symbolic view is whether language can activate the neural mechanism responsible for perception. However, the existing experimental findings could not answer this question because both theories can explain them. Specifically, the embodied view holds that the interaction between language and perception occurs at perceptual level. However, the symbolic view believes that the interaction occurs at the semantic level. Considering this limitation of previous studies, we used a new paradigm to test these two theories by clarifying whether language comprehension involves the neural mechanisms of the perceptual system.

Notably, adaptation paradigm was often used to investigate the neural mechanisms of perceptual processes. In this study, we made use of one such adaptation measure, color adaptation. Generally, an adaptation paradigm includes two stages. First, in the adaptation stage, participants are exposed to a certain stimulus. Second, after prolonged adaptation, participants are asked to do a perceptual task, so that the aftereffect caused by the adaptation stimulus can be estimated. For example, the motion aftereffect is a phenomenon in which after prolonged exposure to upward motion, people are more likely to see a stationary stimulus as moving downward, and vice versa [20]. This illusion is believed to arise from the adaptation-induced decrease in activity of directionally selective neurons [20,21]. The imbalanced activity of neurons leads to an amplified response in the other direction.

Dlis and Boroditsky [22] made an important change to the adaptation paradigm, using language materials instead of visual objects as adaptation stimuli. They found that after listening to stories describing motion in one direction, participants were more likely to perceive directionally ambiguous stimuli as moving in the opposite direction. Actually, the result provides evidence for the embodied semantics that language processing shares a neural mechanism with the perception of real stimuli, as the author stated that high-level cognitive processing is grounded in the perceptual-motor system.

Here we made use of color adaptation to investigate the effect of language on color perception. According to color perception theories, there are color-selective neurons in the cortex that are sensitive to certain colors [23,24]. For example, when people see a red object, the neurons representing red will be more easily activated than the neurons representing green. Then, the selective adaptation of neurons tuned to a certain color (e.g., red) would result in relatively stronger activity of neurons tuned to its complementary color (e.g., green) in the adapted region. This is somewhat like the motion aftereffect mentioned above.

In this experiment, the language stimuli were discourses depicting red, green or non-specific color sceneries (neutral discourse). We tested whether color-related language adaptation could cause color aftereffect. The typical color aftereffect is color afterimage. Previous studies often tested the duration of afterimage to assess perceptual color aftereffect [25]. However, it is very hard to induce afterimage after listening to color-related sentences, let alone estimate its duration. So we have to develop another way to assess the linguistic adaptation color aftereffect. Given that aftereffects actually lead to a change in sensitivity, we focused on the sensitivity difference to a certain color after exposing to different linguistic contexts. The detection task was used to assess sensitivity change. After listening to a few sentences, participants needed to decide whether they saw a green color square in the middle of the screen. There were three conditions according to the relation between the color of the language stimuli (discourses) and the visual stimuli (green color squares): complementary (“red” discourse, green square), congruent (“green” discourse, green square), unrelated (neutral discourse, green square). The embodied view predicts that understanding linguistic descriptions of color recruits the same neural networks as involved in color perception, so that prolonged exposure to a color linguistic context will cause an adaptation effect to improve the sensitivity of its complementary color. Consequently, participants will be more likely to perceive the green color square after listening to the red discourse than the green or neutral discourse. By contrast, symbolic semantics hold that comprehension is independent of neural networks involved in perception, so there should be no significant difference across conditions or a semantic priming effect that the green color square will be more likely to be perceived in the congruent condition than the complementary or unrelated condition. The later hypothesis is based on the view that language will prime the perceptual process at the semantic level [18].

The results of the detection task could be calculated with Signal Detection Theory [26], so that we could assess whether and at which level language comprehension affects color perception. And the effects those at the perceptual level (sensitivity) and the decision level (criterion) could be separated. If language comprehension activates the earliest stage of sensory processes, we would expect an effect on sensitivity. If language comprehension acts only on high-level processes, we would expect an effect on the decision criterion, but not sensitivity [27]. Based on the above analysis, we can test the embodied view and symbolic view of language by investigating the influence on color perception from the adaptation induced form language stimuli.

## Method

### Ethics statement

The current study was approved by the Academic Committee of the School of Psychology at South China Normal University. All participants gave written informed consent before participating in the experiments.

### Participants

Thirty-eight students (23 females, 15 male) from South China Normal University participated in exchange for a small payment. All of them were native Chinese speakers. Participants had normal or corrected-to-normal visual acuity and were naive to the purpose of the experiment. All had binocular vision and binaural hearing. No participants reported having dyslexia or any problems with distinguishing colors.

### Adaptation stimuli

Nine texts depicting sceneries were used. They were broken up into one longer and five shorter “top-up” installments so that multiple measurements could be collected. The first paragraph was long, including about one hundred Chinese characters, and the other 5 paragraphs were short, including about fifty Chinese characters. Then the texts were converted into sound, with the first paragraph lasting 30 seconds or so, and others about 15 seconds. The audio recording was made of a female student who was blind to the color manipulation. The audio files were presented via closed stereo headphones.

According to the color being described, the nine discourses were divided evenly across three types: red (e.g., sunrise), green (e.g., bamboo forest), and neutral (e.g., spring tide). For detail, see the appendix.

### Test stimuli

Following each discourse installment, participants indicated whether they saw a green color square in the middle of the white screen. All experiments were carried out on the same computer. The distance between the subjects’ eyes and the screen was fixed at 60 cm. A chin rest was used to maintain viewing distance. Test stimuli were presented on a 17-inch cathode-ray tube monitor with a resolution of 1024×768 pixels and a refresh rate of 60 Hz. The display adaptor software is 32-bit color. The monitor's color temperature was set to 9300K. The Contrast was set to maximum and the Luminance was 100 cd/m2. The monitor gamma is 2.2.

Six green color squares were used as text stimuli, which could be divided into small and large groups according to the visual angle: 5.0×5.0, 2.7×2.7 degrees; Each group included three squares which differed on the alpha value: 1% (small 1, large 1), 2% (small 2, large 2), 3% (small 3, large 3). The alpha channel is independent of the RGB channel, it was introduced by Alvy Ray Smith in the 1970s. Alpha value is a measure of opacity and ranges from 0%-100%. Values between 0% and 100% make it possible for pixels to show through a background like a glass. If a pixel has a value of 0% in its alpha channel, it is fully transparent (and, thus, invisible), whereas a value of 100% in the alpha channel gives a fully opaque pixel. The alpha channel allows easy image compositing. In this study, it allows us to make light green squares.

The green squares were presented in a white background, lower the alpha value was, the square looked less green and was harder to be detected. It should be noted that the alpha values used here are very low, so the light green squares being used were not easy to be detected. The average accuracy to detect them was about 50%, close to the thereshold. Besides green squares, blank white screen without squares on it was also being tested. Therefore, we had seven types of test stimuli: small or large green color squares of three alphas, and the white screen. We first set the RGB values to 0, 255, 0 to get pure green squares, and also modulated the alpha value of each square. The RGBA value of each square are as follows: small 1/large 1 = 0, 255, 0, 0.01; small 2/large 2 = 0, 255, 0, 0.02; small 3/large 3 = 0, 255, 0, 0.03; the white background = 255, 255, 255, 1.

Because the green squares were displayed in a white background, so we composited the green square with the white background. And we used this compositing images as test stimuli. The RGB values of each compositing square are as follows: small 1/large 1 = 252, 255, 252; small 2/large 2 = 250, 255, 250; small 3/large 3 = 247, 255, 247; the white background = 255, 255, 255. After the experiment, we determined coordinates of color stimuli in the CIE L*a*b* color space by using the conversion software at www.easyrgb.com, with parameter set to illuminant daylight and observe 2 degrees. The CIE L*a*b* values of each green square are as follows: small 1/large 1 = 99.707, -1.507, 1.073; small 2/large 2 = 99.513, -2.516, 1.799; small 3/large 3 = 99.225, -4.029, 2.892; the white background = 100.000, 0.005, -0.010.

There were 3 discourses with 6 installments in each condition, so that 18 green squares were used as test stimuli in one block. The frequency of each test stimulus used in one condition is shown in Table 1. Reasons for us to choose the current frequencies are as follows: firstly, if we only use one kind of stimulus, participants would soon learn to respond to the test stimuli expertly, which would lead to practice effects. Secondly, different kinds of stimuli could make the participants stay alert so that to avoid the potential fatigue effect. Two previous studies [22,28] concerning motion also used several kinds of test stimuli. Thirdly, we used signal detection theory (SDT) to calculate the accuracy data, so most of the test stimuli should be sufficiently difficult yet discriminable for participants. And comparing with 3 alpha squares, 1% or 2% alpha value squares could better meet the requirement. Therefore, most of the test stimuli were 1% or 2% alpha value squares. Lastly, we used the SDT, so the test stimuli included blank stimuli for calculating the false alarm rate.

**Table 1:** Frequency of each test stimulus.

	0	1%	2%	3%
Small (2.7×2.7)	4	3	3	1
Large (5.0×5.0)		3	3	1

### Design and procedure

The experiment was a one factor (the relation between the color of the discourse and the square: complementary, congruent, unrelated) within-subject design. Three types of discourses (red, green, neutral) were presented in three interleaved blocks. Each block consisted of three discourses with six installments each, for a total of 18 trials per block. A trial was defined as a single presentation of the adapting stimulus followed by a single presentation of a color square or a white screen.

Participants were asked to close their eyes when listening to the discourse. Following each installment, they heard a short sound “ding” and opened their eyes immediately to indicate whether or not they see a green color square in the middle of the screen by pressing one of the designated keys (“F” corresponds to “Yes” and “J” corresponds to “No”) on a standard keyboard. If they made no response within 2500ms, the test window would disappear.

Before the formal experiment, there was a practice phase. The whole experiment lasted about 20 minutes. The orders of blocks and the orders of discourses within each block were balanced for each subject. No participants reported knowledge of color adaptation, motion aftereffect, and similar phenomena.

### Data analysis

Participants were excluded from analysis if the hit rate or false alarm was beyond 2.5SDs above or below the average. Finally, data from 32 participants (19 females, 13 male) were included in subsequent analysis. 9 trials (1.56% of the total) were deleted for making no response. The data were submitted to a one-way repeated measure ANOVA using SPSS 17.0.

The dependent measures were color-detection accuracy (SDT measure) and RT. It should be noted that in order to rule out an RT/accuracy tradeoff we used the median RT (in case there were outliers on the long end). Results were analyzed for three conditions: complementary (red discourse, green square), congruent (green discourse, green square), unrelated (colorless discourse, green square).

For SDT analysis, we used the accuracy data to calculate the percentage of “Yes” responses (correct detections) on green color square trials (hits) and the percentage of “Yes” responses (incorrect detections) on white screen trials (false alarms). The nonparametric indexes of perceptual sensitivity, A’, and response criterion, B”, were calculated from hit and false alarm rates. Calculations were performed in Excel using equations taken from Stanislaw and Todorov [26]:

$$\text{A'}=\left\{ \begin{array}{l} 0.5+\frac{\left(\text{H}-\text{F})+(\text{1}+\text{H}-\text{F}\right)}{4\text{H}(1-\text{F})}\text{when H}\geq \text{F}\\ 0.5-\frac{\left(\text{H}-\text{F})+(1+\text{F}-\text{H}\right)}{4\text{H}(1-\text{F})}\text{when H}\leq \text{F} \end{array}\right.$$

$$\text{B''}=\left\{ \begin{array}{l} \frac{\text{H}(1-\text{H})-\text{F}(1-\text{F})}{\text{H}(1-\text{H})+\text{F}(1-\text{F})}\text{when H}\geq \text{F}\\ \frac{\text{F(1}-\text{F)}-\text{H}(1-\text{H})}{\text{F}(1-\text{F})+\text{H}(1-\text{H})}\text{when H}\leq \text{F} \end{array}\right.$$

## Results

First, we calculated the mean accuracy and median reaction times of each test stimulus, as Tables 2 and 3 show. Table 4 shows the mean accuracy for each level of transparency. Considering the limited number of each test stimulus, further analysis was carried out over all the test stimuli within each condition.

**Table 2:** Mean accuracy of different color squares in three conditions (n = 32).

size	blank	small			large			average
alpha	0	1	2	3	1	2	3	
complementary	94.5%	71.9%	86.5%	90.6%	17.7%	62.5%	81.3%	60.6%
congruent	85.0%	62.1%	82.3%	90.6%	20.8%	57.9%	87.5%	58.6%
unrelated	88.3%	58.3%	84.4%	93.8%	15.6%	68.7%	78.1%	58.3%

**Table 3:** Mean median response times (ms) of different color squares in three conditions (n = 32).

size	blank	small			large			average
alpha	0	1	2	3	1	2	3	
complementary	1002.2	971.8	909.2	916.5	1058.2	1016.1	1125.2	995.3
congruent	1019.2	1022.8	903.3	917.5	1071.2	1023.8	1033.6	1001.7
unrelated	1031.0	978.5	969.4	867.1	1076.8	1004.3	1006.5	1005.1

**Table 4:** Mean accuracy for each level of transparency in three conditions (n = 32).

alpha	0	1	2	3
complementary	94.5%	44.8%	74.5%	85.9%
congruent	85.0%	40.6%	50.7%	54.9%
unrelated	88.3%	37.0%	76.1%	85.9%

**Fig 1:**
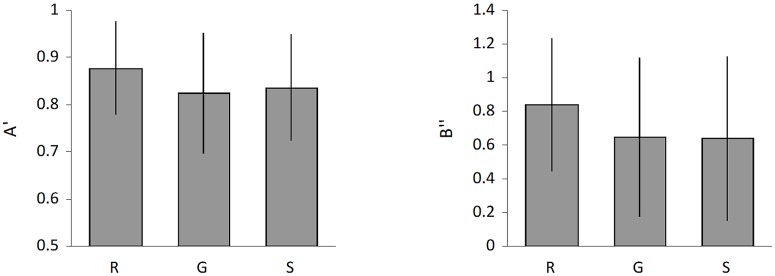
Results of A’ and B”. “R” means red discourse, representing the complementary condition. “G” means green discourse, representing the congruent condition; “S” means neutral discourse, representing the unrelated condition.

The means of the hit rate and false alarm estimates are given in Table 5. The means of the resulting A’, B” estimates are given in Table 6. The one-way repeated measure yielded a main effect for A’ and B”, see Fig 1. For RT, there was no significant difference across conditions.

**Table 5:** Estimates of P(hit) and P(false) in three conditions (n = 32).

	P(hit)			P(false)		
	complementary	congruent	unrelated	complementary	congruent	unrelated
M	0.63	0.60	0.61	0.05	0.15	0.12
SD	0.19	0.17	0.19	0.14	0.22	0.17

**Table 6:** Estimates of A’ and B” in three conditions (n = 32).

	A’			B”		
	complementary	congruent	unrelated	complementary	congruent	unrelated
M	0.88	0.82	0.84	0.84	0.64	0.64
SD	0.10	0.13	0.11	0.39	0.47	0.48

**Note**. Higher A’ values indicate a better discrimination ability, and higher B” values indicate a relative bias to response “No,” whereas smaller B” values indicate a relative bias to response “Yes”.

### A’

The relation between the color of the discourse and the square significantly modulated perceptual sensitivity for green color square. There was a significant main effect of condition [*F* (2, 62) = 4.00, *p* = 0.024, *ŋ*_*p*_^2^ = 0.113]. Pairwise comparison revealed a significant difference between the complementary (*M* = 0.877, *SE* = 0.02) and congruent condition (*M* = 0.824, *SE* = 0.02) (*p* = 0.019), and a significant difference between the complementary and unrelated condition (*M* = 0.836, *SE* = 0.02) (*p* = 0.031). There was no significant difference in A’ between the congruent and unrelated condition (*p* = 0.550). The estimated difference of mean A’ between the complementary and congruent condition was 0.05, 95% CI [0.01, 0.10], 0.04, 95% CI [0.00, 0.08] between the complementary and unrelated condition, and -0.01, 95% CI [-0.05, 0.03] between the congruent and unrelated condition. Overall, participants were more sensitive to green color square after listening to the discourses denoting red than after listening to the discourses denoting green or non-specific color information. In other words, listening to discourses denoting a certain specific color improved the sensitivity of its complementary color.

### B”

For B”, there was a significant difference across conditions [*F* (2, 62) = 3.72, *p* = 0.030, *ŋ*_*p*_^2^ = 0.107]. Pairwise comparison revealed a significant difference between the complementary (*M* = 0.837, *SE* = 0.07) and congruent condition (*M* = 0.644, *SE* = 0.08) (*p* = 0.025), and a significant difference between the complementary and unrelated condition (*M* = 0.637, *SE* = 0.09) (*p* = 0.025). There was no significant difference in B” between the congruent and unrelated condition (*p* = 0.930). The estimated difference of average B” between the complementary and congruent condition was 0.19, 95% CI [0.03, 0.36], 0.20, 95% CI [0.03, 0.37] between the complementary and unrelated condition, and 0.01, 95% CI [-0.16, 0.18] between the congruent and unrelated condition. The significant B” suggested that response bias affected the reaction to the test stimuli.

### RT

There was no significant difference in median RT across conditions (*p* = 0.955). The estimated difference between the mean median RT of the complementary (*M* = 995.35, *SE* = 51.16) and congruent condition (*M* = 1001.68, *SE* = 49.84) was -6.33, 95% CI [-80.25, 67.59]. -9.73, 95% CI [-80.77, 61.32] between the complementary and unrelated condition (*M* = 1005.08, *SE* = 54.63), and -3.40, 95% CI [-55.81, 49.01] between the congruent and unrelated condition.

## Discussion

This study is the first one to make use of color aftereffect to investigate the influence of language comprehension on color perception within the embodied cognition framework. Sentences denoting color-related information were followed by a color square detection task. We found that listening to discourses denoting a certain color (red) improved the sensitivity to its complementary color (green) and also influenced the internal decision criterion, but not the speed in performing a color detection task. The results found here were consistent with a previous study done by Meteyard, Bahrami, & Vigliocco [27], but for motion language instead of color language.

A’ is a sensitivity index of SDT, higher A’ means better discriminability. The results showed that A’ was higher when the color of the sentences and the test square complementary to each other (red, green) compared to the congruent or unrelated condition, suggesting that listening to sentences denoting a certain color (red) will improve the sensitivity to its complementary color (green). This linguistic adaptation color aftereffect could be compared with the perceptual adaptation color aftereffect that after looking at a red object for minutes, people will perceive a green afterimage on a white background. Both may activate the neural basis responsible for color perception and cause a neural adaptation. Thus, we can deduce that listening to a discourse describing red is like seeing the real red visual object. This is in accordance with the hypothesis of simulation view of embodied language, which believed that language comprehension is constructing mental simulations of the things being described, and these simulations will re-activate the sensorimotor systems responsible for real perception and action.

B” is a response bias index of SDT, higher B” suggesting a higher criterion, which signifies that the participant is being more conservative and have a bias to respond “no” [26]. We found an upward shift B” in the complementary condition than the congruent or unrelated condition, which indicated that at the decision stage, participants would be more likely to respond “I didn’t see a green color square” after hearing “red” sentences. In other words, the color of the sentences primed participants’ reactions to the perceptual stimuli of the same color. The result of B” revealed a match advantage effect (priming effect) at the decision stage, which was consistent with the hypothesis of the symbolic semantics. However, the symbolic view could not account for the result of A’, because the symbolic semantics hold that language comprehension acts only on high-level processes, but not the early stage of sensory processes. Therefore, an effect on the decision criterion would be expected, but not sensitivity. However, the embodied semantics predict a complementary advantage effect on low level perceptual sensitivity, which had been proved by the higher A’ in the complementary condition, and the effect on decision (B”) was a consequence of the change in sensitivity. For example, in the previous study [25], researchers suggested that the priming effect on B” could be accounted for by task-based expectations: color sentences created an expectation of the particular color and this expectation influenced processing generally, which resulted in priming at the decision level.

To sum up, the traditional symbolic semantics could not explain the current result that language comprehension infected the early stage of perceptual processes. The results of the perceptual sensitivity (A’) and the internal decision criterion (B”) could be better explained by the embodied semantics which predict that language comprehension affected the low level perceptual system regardless of the task-induced change at the decision stage.

It should be noted that neither A’ nor B” was significant between the congruent and unrelated condition. This result was beyond our expectation that a “green” discourse should make for a lower sensitivity rather than being equal to the effect of the neutral discourse. The possible reason was that the non-specific color discourse lent the participants more toward green imagery than red. For example, in the “spring tide” text, people might be more likely to imagine lush greenery around the river depicted by the story than they would be to imagine red elements. This could be why the “green” and “colorless” stimuli pattern similarly. This is a possible reason, but in the study of Meteyard et al [27], they also found that there was no significant difference between the congruent and neutral condition, so there may exited other reasons responsible for this phenomenon. Further studies were needed to explore this problem.

The reaction times were not significantly different across conditions, which was basically consistent with our expectations. On one hand, purpose of our study was to investigate whether the sensitivity to a certain color would be meditated by linguistic stimuli. What we concerned was whether and to what extent they could perceive the green square but not how fast they making a response. Therefore, in this study, we attached more importance to SDT indexes, which were also used in the former study done by Pavan and Baggio [18] to investigate the effect of motion words on direction discrimination task. On the other hand, participants were asked to close eyes when listening to the sentences, and open eyes when heard the sound “ding” and then make a response. It should be noted that in each trial, the timing they opening eyes and the time needed from opening eyes to making a response varied, which would definitely influence the reaction times (from the onset of the test window to the start of making a response). Taking this into consideration, the RT data could not reliably reflect the effect of experimental treatments.

The language adaptation method is an improvement of the previous prime paradigm (a word or a sentence as a priming stimulus). The biggest difference was longer exposure to linguistic stimuli which provided the possibility of neural adaptation, as being revealed by Dlis and Boroditsy [22]. The results found here put further evidence for the previous match advantage effect [16,29]. The defect of the match advantage effect was that it could be explained not only at the perceptual level but also at the semantic level. However, the adaptation aftereffect found here could only be explained at the low level of perceptual system or neural networks. Thus, we can deduce that the interaction between language and color perception occurs at the perceptual level, which is the focus of the debate between the embodied view and the symbolic view. Furthermore, the adaptation aftereffect could be seen as a mismatch aftereffect, because A’ decreased in the congruent (match) condition compared to the complementary (mismatch) condition. However, it was different from the previous mismatch advantage found by Kaschak et al [12]. In their study, the sentence and the visual stimulus were presented concurrently. The task was determining the motion direction indicated by the sentence. And a mismatch advantage was found because the perceptual stimulus engages the processing mechanisms needed to simulate the sentence, which resulted in comprehension difficulty when the content of the sentence matched the content of the stimulus [12]. In the present study, sentences and color squares were presented sequentially, which ruled out the possibility of cognitive resource competition.

As far as we know, two other studies also used the adaptation paradigm to investigate the relations between language comprehension and perception, and both focused on motion aftereffects. Dlis and Boroditsky’s results [22] were consistent with the embodied semantics as were our results. However, Pavan and Baggio [18] used a visual-adaptation paradigm to test whether the perceptual adaptation would influence the language comprehension and found a match advantage effect which was contrary to the embodied view. It seems like that the adaptation caused by language does influence motion perception, but not vice versa. Further studies are needed to investigate this problem. However, from our point of view, in Pavan’s experiments, participants were required to judge the motion direction of the verb phrase as soon as possible; thus, there existed a possibility that in this rapid language processing condition, participants had no time to use the sensorimotor information to construct mental simulation. So the verb phrases were processed at the semantic level and were not influenced by the motion aftereffect. In this way, the results perhaps demonstrate that in the case of rapid language processing condition, semantic information is more preferable than sensory information in constructing the meaning of language. If the deduction could be further confirmed, we can propose a theoretical assumption that: in general, language comprehension reactivates the sensorimotor system. Whereas, in the case of rapid language processing, individuals taking advantage of the existing semantic but not perceptual information in the first place to gain the meaning of language. To be brief, language comprehension will activate the perceptual information automatically, but the perceptual information will not facilitate the process of language in an automatic way. Of course, this is a preliminary idea, and a series of experiments would be required for verification.

There are limitations in this study worth mentioning. First, only green squares were used as test stimuli. It would lead to a potential problem that the observed color aftereffect was not caused by the color information implied in the discourse but other potential factors indicated by the discourses. A better way to avoid this problem was using both green and red squares as test stimuli. However, in this study, considering the formation of color adaptation process would be disturbed, we only used green squares. To avoid the potential problem, we carefully controlled the discourses being used when designing materials. For example, the number of Chinese characters, sentences, and color words was controlled. The contents of the discourses were all about sceneries. And all of the texts were read by a subject who did not know anything about the main experiment. In this way, the interference factors could be excluded to a large extent. In the field of color perception, red adaptation could cause green afterimage, and in turn, green adaptation could cause red afterimage. Then, this should also be true for the linguistic color aftereffect that red context should causes sensitivity increase for green square and green context should in turn causes sensitivity increase for red square. In future studies, this problem should be taken into serious consideration, such as dividing the experiment in green and red adapter sessions. In this study, we have tried our best to control the irrelevant variables of the discourse, so we could make a relative strong claim that the effect found was actually a color aftereffect.

The second limitation of this study is that we didn’t calibrate the monitor in a very strict way. Computer generated colors are often deeply affected by the monitor characteristics. So even though we provide the RGB values and the CIE coordinates of the stimulus, it is not enough. The monitor should be calibrated before starting the experiment, otherwise the color deviation will make the results less credible and harm the reproducibility of the experiment. The flaw is that we calibrated the monitor only by naked eyes instead of using professional calibration tools, this maybe too arbitrary and subjective, because accurate color-matching call for professional calibration. However, considering that we did actually controlled the contrast, luminance, color temperature of the monitor, and all the experiments were carried out on the same computer, which means the gamma value, resolution and other parameters of the screen stay the same. In this way, the color deviation could be minimized to a large extent. Further more, our research topic is color afterimage, previous study [30] has shown that the afterimage perceived was roughly, not exactly complementary to the hue of the color perceived during adaptation. And the afterimage itself is not stable, the strength, brightness and duration varies a lot [31]. Therefore, we believe that the color aftereffect founded in this study is not so vulnerable to small color deviation of the test stimuli. In sum, the color deviation may exist, but it has little effect on the results. However, future study should pay more attention to monitor calibration to avoid potential problems.

The third limitation was lack of discussion about whether the linguistic adaptation color aftereffect tends to build and strengthen with exposure to the adapting stimuli. In Dlis and Boroditsky’s [22] research of the language motion aftereffect, they found no aftereffect when participants finished listening to the first two paragraphs of a story, but after participants finished listening to the whole story, the results showed a motion aftereffect. Thus, further studies could concentrate on the addictive feature of adaptation aftereffect in order to assure that the language-induced color aftereffect was caused by the same process—neuron adaptation—as the perceptual color aftereffect. Another interesting question is whether the aftereffect resulted from the whole text (narrative level) or the color words (lexical level). That is to say, whether a single word independent of the context could activate the simulation mechanism?

On the whole, we made use of the linguistic adaptation paradigm instead of the previous match-mismatch paradigm to investigate the influence of language comprehension on color perception. Our study made up the insufficiency of the previous studies on simulation theory about color. The linguistic adaptation color aftereffect revealed here is different from the previous match or mismatch advantage effect and provides more powerful support for the embodied semantics that language comprehension and perceptual processing share, at least to some extent, the same neural mechanism. Our study also has important implications in terms of methodology. The linguistic adaptation paradigm proposed here provides a new means to examine the effect of language processing on perception, so that we have a more powerful method to test whether the neural bases of conceptual and perceptual processing are overlapped. As such, it might also be promising to expand the paradigm for studying the comprehension of words or sentences conveying other perceptual information.

## Appendix

### Texts (six installments each):

#### Red texts:

**Sunrise:**
1. See from the distance, there appeared red color on the horizon and gradually became deeper and deeper, spreading in a wide range and thus turning the clouds nearby into red color. The sky, the mountain range and the branches all seemed to be dressed in red brocade and that was rosy dawn. Gradually, the sun showed up in the shape of half round with brightly red color.
2. The red, half-round sun rose continuously. The color of the sun was deep red, like a shining bloody red plate of agate.
3. Finally, the red sun became rounder and rounder and rose over the horizon. As red and powerful as fire, the sun came out.
4. The light of the sun spreads around, so there appeared thousands of rosy clouds, making the clouds nearby dyed in magnificent red color.
5. The expanse earth was smeared with a layer of brightly red greasepaint and all things on earth were dressed in splendid attire.
6. Red trees, red chicken and red houses were all under the light of the rosy dawn, the red color of which was really adorable.

**Maple trees:**
1. Walking along the mountain path and all of a sudden, a large piece of red chunk came into sight, like a ball of burning fires, sparkling in the sunlight, which is extremely beautiful! Take a closer look, they are maple trees. Mountains have a winding distribution and were covered with red maple leaves, like an immense red cloth spreading out on the ground.
2. The whole hillside is covered by the brightly burning maple trees. Looking from a distance, it seems like the mountain is flaming.
3. All the maple trees together are like a cluster of flames with scarlet color. They are burning, leaping and dancing in the wind.
4. The color of the dancing leaves is red. They are so bright and hot, like fireballs. The red color makes people excited and heartbeat.
5. The maple leaves are hot red as fire, fresh and bright. Dancing and waving in the autumn wind. it seems like they are boasting themselves.
6. With red leaves, the gleam of sunny clouds is dazzling as splendid brocade. It is the most charming warm color in the cool autumn.

**Azalea:**
1. Azalea, also called rhododendron, has a reputation of “the beauty of flowers”. The petals of azalea are red. They are next to each other, pushing and pressing, how lovely! When standing in front of ten thousands mu of azaleas. You can see fiery-red azaleas blooming all over the slope. The land is like being coated with a layer of rouge.
2. The red color of the boundless azalea bushes is fervent, solemn and stirring. It looks like the red was going to stretch to the horizon.
3. See from a distance, the wall of red flowers dying the sky red. Flaming red flowers covered the branches, bravely waving in the wind.
4. Each blooming azalea is like a red agate, fluttering in the wind. And exposure in the sunlight makes them more bight and vivid.
5. There could be no other flowers blooming so overwhelmingly like the azaleas do, they make every place of the mountain slope red.
6. The flower sea is like flaming red rosy clouds or a burning torch. The fire-alike tide of flowers surged in the lonely mountain.

#### Green texts:

**Green bamboo forest:**
1. From the flatland to distant mountains, all lands are covered with green bamboos. The bamboos’ green branches, stems and leaves can’ t be distinguished from each other. The houses, pathways and running water under the small bridges are all covered by the sea of bamboos. When there is wind blowing, the green bamboo sea surges, wave after wave, rushing to the distance.
2. It is difficult to learn how deep the green bamboo sea is, but the fluctuation of the wave and its power showed its depth.
3. A light rain comes in the mountain, breaking the previous quietness. Looks like the bamboos are converted into a clump of light green.
4. The rain drops on the green leaves, making a sound of rustle. The stretches and leaves indulged in the sweet dew of spring.
5. Bathing in the drizzle, the bamboos become greener and greener, changing its color from light to dark, making you feel relax and happy.
6. After raining, the bamboos appear refresher and greener. The leaves are like flawless jades and the forest resembles a big green protective screen.

**Spring is coming:**
1. Open the window, the world outside is really a surprise. It is a world of green, an ocean of spring. There are light green ivies, covering the entire wall with their verdant leaves. And the tiny, green grasses emerge from the soil quietly, and blanket the fields throughly. Thousands of miles are green. See from a distance, the land looks like a green cotton blanket.
2. The green wheat seeding field in the distance changes its color in the sunshine from dark green, blue green to light green.
3. The pond is also green, it is a mixture of summer’s verdant, autumn’s gloss green and winter’s green black, deep and unpredictable.
4. As if a green ink bottle was spilled, everywhere is green. There are so many green colors: dark green, light green, pale green, pink green……
5. The green color is deep and interesting. All the Leaves are green, grasses are green, lake water is green and the raindrops are also green.
6. All the green colors gather together, pressing and crossing. A sudden gust of wind makes all of them dancing neatly to the beat.

**Green hills and waters:**
1. Sitting in a boat ridden on the lake among the green water and mountains, I am surprised by the green color in front of eyes. The water is so clear that the stones can be seen clearly. The water is so green, pure and lovely, as a flawless jade. The surface of the lake ripples when gentle breeze blows. The green water goes appropriate with the mountains.
2. The mountains have different green colors. Near view, they are light green. A littler far away was jade green. More far away was dark green.
3. As if the green color of the verdant mountain was dyed a moment ago. All of the several fresh trees are with tender green leaves.
4. The rain seems to stop but not, dropping into the world. It seems like the rain is also green, softly shed from the sky.
5. In the dense spring rain, all the green color is vibrating, with a glittering green light. Everything is so fresh, peaceful and poetic.
6. Standing here, with green mountains surrounding the water, and green water reflecting the mountain. Feels like walked into a continuous green oil painting.

#### Non-specific color texts:

**Winter in Jinan:**
1. Please close your eyes and imagine a scenario to yourself: an ancient city, with mountains and lakes, all of them are dozing in the sunlight, warm and comfortable, waiting for the spring breezes to blow it awake. Isn’t it an ideal state? Low hills practically encircle Jinan, leaving just one small opening at the north of Jinan.
2. The endearing hills cradle the city and murmurs: “Don’t worry, it is warm here”, giving people a place to rely on.
3. Looking up and around, people say to themselves: “Maybe spring will come tomorrow, maybe tonight the grass on the hills will sprout.”
4. And even if this imagination can’t come true, they don’t really mind, because with such kindly winter, what else will they expect for?
5. A light snow makes the scene even prettier. The small pines on the hills become darker and blacker. The peaks are like a silver edge on the azure sky.
6. The snow that is on the slope, somewhere is thick, and somewhere the grasses are still exposed; Looks like the hill is wearing flowery clothes.

**The lotus pool by moonlight:**
1. All over this winding stretch of water, what meets the eye is a silken field of leaves, reaching rather high above the surface, like the skirts of dancing girls in all their grace. Here and there, layers of leaves are dotted with white lotus blossoms, some in demure bloom, others in shy bud, like scattering pearls, or twinkling stars, or beauties just out of the bath.
2. The moon sheds liquid light over the leaves and flowers, and a floating light mist makes them seem been bathed in milk.
3. Though the moon is full, the light is not bright; but it is just right–a profound sleep is indispensable, vet short naps have their own charm.
4. The moonlight is streaming through the foliage, casting shadows on the ground, as grotesque as spectre, and the willows like paintings on the lotus leaves.
5. Around the pond, far and near, high and low, are trees, which have the pool entirely hemmed in, the only clearings left being those by the path.
6. Trees are somber as dense smoke. Above the tree-tops loomed distant hills. Through the branches are a couple of lamps, as listless as sleepy eyes.

**The Qiantang River tide:**
1. Qiantang River Tide has been called world wonders since ancient times. The annual lunar August 18 is the best time to watch the tide. Early in the morning, we reached Yanguan of Haining City, which is the best place to appreciate the tidal bore. The dam had already been crowded with people before the tidal bore began. People just waited there, raising their heads and looking toward the east.
2. At one O’clock in the afternoon, there was a huge booming sound from the distance. Someone told us that the tide was coming.
3. We looked towards the east. The sound became louder and there appeared a thick line that connected the sky and the water.
4. The line moved forward swiftly, becoming longer and wider, then spreading around the river. When nearer, the line became waves.

## Supporting information

S1 File Original date. (RAR)
