# Correction: Color adaptation induced from linguistic description of color

**DOI:** 10.1371/journal.pone.0224597

**Published:** 2019-10-24

**Authors:** Liling Zheng, Ping Huang, Xiao Zhong, Tianfeng Li, Lei Mo

In the Data analysis subsection of the Method section, there are typographical errors which were introduced into the equation for the nonparametric index of perceptual sensitivity, A’, taken from Stanislow and Todorov.[2] Please see the complete, correct equation here:

$$A'=\left\{ \begin{array}{l} 0.5+\frac{\left(H-F)(1+H-F\right)}{4H(1-F)}\mathrm{when}\;H\geq F\\ 0.5-\frac{\left(F-H)(1+F-H\right)}{4F(1-H)}\mathrm{when}\;H<F \end{array}\right.$$
